# Disruption of the RICTOR/mTORC2 complex enhances the response of head and neck squamous cell carcinoma cells to PI3K inhibition

**DOI:** 10.1002/1878-0261.12558

**Published:** 2019-08-28

**Authors:** Kara M. Ruicci, Paul Plantinga, Nicole Pinto, Mohammed I. Khan, William Stecho, Sandeep S. Dhaliwal, John Yoo, Kevin Fung, Danielle MacNeil, Joe S. Mymryk, John W. Barrett, Christopher J. Howlett, Anthony C. Nichols

**Affiliations:** ^1^ Department of Otolaryngology – Head and Neck Surgery, Schulich School of Medicine & Dentistry Western University London Canada; ^2^ Department of Pathology & Laboratory Medicine, Schulich School of Medicine & Dentistry Western University London Canada; ^3^ Department of Oncology, Schulich School of Medicine & Dentistry Western University London Canada; ^4^ Department of Microbiology and Immunology, Schulich School of Medicine & Dentistry Western University London Canada

**Keywords:** head and neck cancer, mTORC2, PI3‐kinase, RICTOR, targeted therapy

## Abstract

Phosphoinositide 3‐kinase (PI3K) is aberrantly activated in head and neck squamous cell carcinomas (HNSCC) and plays a pivotal role in tumorigenesis by driving Akt signaling, leading to cell survival and proliferation. Phosphorylation of Akt Thr308 by PI3K‐PDK1 and Akt Ser473 by mammalian target of rapamycin complex 2 (mTORC2) activates Akt. Targeted inhibition of PI3K is a major area of preclinical and clinical investigation as it reduces Akt Thr308 phosphorylation, suppressing downstream mTORC1 activity. However, inhibition of mTORC1 releases feedback inhibition of mTORC2, resulting in a resurgence of Akt activation mediated by mTORC2. While the role of PI3K‐activated Akt signaling is well established in HNSCC, the significance of mTORC2‐driven Akt signaling has not been thoroughly examined. Here we explore the expression and function of mTORC2 and its obligate subunit RICTOR in HNSCC primary tumors and cell lines. We find RICTOR to be overexpressed in a subset of HNSCC tumors, including those with *PIK3CA* or *EGFR* gene amplifications. Whereas overexpression of RICTOR reduced susceptibility of HNSCC tumor cells to PI3K inhibition, genetic ablation of *RICTOR* using CRISPR/Cas9 sensitized cells to PI3K inhibition, as well as to EGFR inhibition and cisplatin treatment. Further, mTORC2 disruption led to reduced viability and colony forming abilities of HNSCC cells relative to their parental lines and induced loss of both activating Akt phosphorylation modifications (Thr308 and Ser473). Taken together, our findings establish RICTOR/mTORC2 as a critical oncogenic complex in HNSCC and rationalize the development of an mTORC2‐specific inhibitor for use in HNSCC, either combined with agents already under investigation, or as an independent therapy.

## Graphical abstract

While the role of phosphoinositide 3‐kinase (PI3K)‐activated Akt signalling is well established in head/neck squamous cell carcinogenesis, the significance of mammalian target of rapamycin complex 2 (mTORC2)‐driven Akt signalling has not been thoroughly examined. Our study reveals an oncogenic role for mTORC2 and its obligate subunit RICTOR. Disruption of RICTOR/mTORC2 signalling impairs cellular viability and enhances the efficacy of PI3K targeted inhibition. Collectively, these findings rationalize the development of an mTORC2‐specific inhibitor.

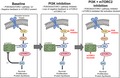

**Abbreviations**
bp: base pair
BYL719: Alpelisib
CDK4/6: cyclin‐dependent kinases 4/6
cDNA: complementary DNA
co‐IP: co‐immunoprecipitation
CRISPR: clustered regularly interspaced short palindromic repeats
E5: exon 5
EGFR: epidermal growth factor receptor
HER2: human epidermal growth factor receptor 2
HNSCC: head and neck squamous cell carcinoma
HPV: human papillomavirus
IHC: immunohistochemistry
mTOR: mammalian target of rapamycin
mTORC1: mammalian target of rapamycin complex 1
mTORC2: mammalian target of rapamycin complex 2
NDGR1: N‐myc downstream regulated 1
PDK1: 3‐phosphoinositide dependent kinase 1
PI3K: phosphoinositide 3‐kinase
PRAS40: proline‐rich Akt substrate of 40kDa
qRT‐PCR: quantitative reverse transcription PCR
RAPTOR: regulatory associated protein of mTOR
RICTOR: rapamycin‐insensitive companion of mTOR
S6K: ribosomal protein S6 kinase
Ser: serine
SGK1: serum/glucocorticoid regulated kinase 1
sgRNA: single‐guide RNA
TCGA: The Cancer Genome Atlas
TCPA: The Cancer Proteome Atlas
Thr: threonine
TMA: tissue microarray

## 1 Introduction

Activating Akt phosphorylation drives cell proliferation, motility, survival, and protein synthesis in numerous cancers, including head and neck squamous cell cancer (HNSCC), which affects over 550 000 individuals worldwide each year (Jemal *et al.*, 2011; Martini *et al.*, 2014). In HNSCC, both human papillomavirus (HPV)‐positive and HPV‐negative tumors show frequent direct activation of Akt, or indirect activation via the phosphoinositide 3‐kinase (PI3K) pathway, which serves as an upstream activator of Akt (Agrawal *et al.*, 2011; Lawrence *et al.*, 2015; Stransky *et al.*, 2011). Accordingly, regulation of Akt is an area of interest for both preclinical and clinical cancer research.

Many studies have focused on PI3K‐PDK1 signaling as the primary means of Akt activation, via phosphorylation of Akt (Thr308) (Engelman, 2009). Inhibition of PI3K blocks Akt phosphorylation at Thr308, leading to decreased downstream signaling to mammalian target of rapamycin complex 1 (mTORC1) and a reduction in cell growth and survival (Engelman, 2009). However, PI3K/Akt/mTORC1 suppression relieves feedback inhibition to upstream network effectors, including mTOR complex 2 (mTORC2), causing a recovery of Akt signaling (Mendoza *et al.*, 2011). As a result of this compensatory signaling adaptation, the efficacy of PI3K pathway inhibition is diminished (Engelman, 2009). To more effectively inhibit Akt signaling in cancer, consideration of these inherent feedback loops is required.

**Figure 1:**
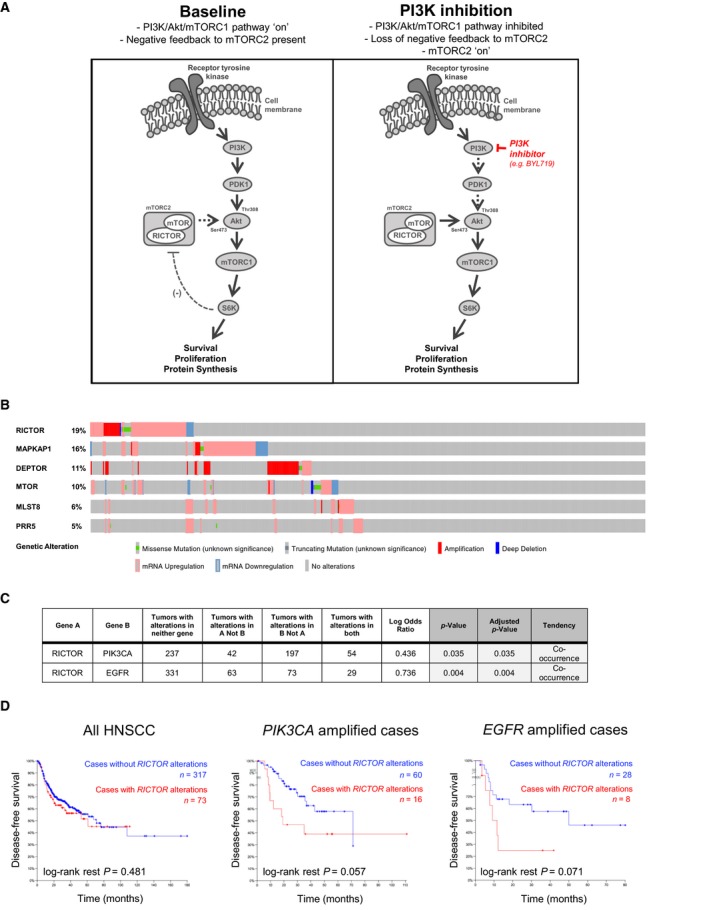
RICTOR/mTORC2 in HNSCC primary tumors. (A) Schematic representation of PI3K/Akt/mTOR signaling cascade with emphasis on negative feedback inhibition of RICTOR/mTORC2 by S6K. (B) Oncoprint showing prevalence of single nucleotide variations (SNV), copy number aberrations, and transcript expression of mTOR complex 2 subunits in TCGA‐curated HNSCC tumors, generated using cbioportal software (https://www.cbioportal.org/). (C) Evaluation of mutual exclusivity or co‐occurrence of genomic aberrations in *RICTOR* and *PIK3CA*, as well as in *RICTOR* and *EGFR* (generated based on TCGA‐curated HNSCC tumors using cbioportal). (D) Kaplan–Meier survival analyses of TCGA‐curated HNSCC cases. Cases were stratified according to the presence or absence of *RICTOR* gene amplification, SNV and mRNA overexpression (> 2 standard deviations above average expression) in HNSCC as whole, or in subsets of HNSCC cases with either *PIK3CA* or *EGFR* amplifications. Cases with *RICTOR* alterations are represented in red.

mTORC2 (formerly known as PDK2) was the second major Akt kinase to be identified and is best known for contributing to Akt activation via phosphorylation of Akt at Ser473(Sarbassov *et al.*, 2005). When PI3K/Akt/mTORC1 signaling is active, the mTORC1 effector p70S6K (S6K) directly phosphorylates the RICTOR subunit of mTORC2 (at Thr1135) to downregulate mTORC2‐mediated Akt activation (Engelman, 2009; Mendoza *et al.*, 2011; Zhang *et al.*, 2016). However, in the case of inhibition of PI3K/Akt/mTORC1 signaling, feedback inhibition of mTORC2 is lost and the complex becomes active (Engelman, 2009; Mendoza *et al.*, 2011; Zhang *et al.*, 2016) (schematic shown in Fig. 1A). mTOR is therefore uniquely positioned to be both activated by Akt (via mTORC1) and to activate Akt (via mTORC2). To date, the importance of mTORC2‐mediated Akt signaling in HNSCC has not be examined in relation to targeting the PI3K pathway.

mTORC1 and 2 are structurally distinct multiprotein complexes, with mTORC1 containing RAPTOR and PRAS40, and mTORC2 containing RICTOR, SIN1, and PROTOR as its distinguishing subunits (Huang and Fingar, 2014; Sarbassov *et al.*, 2004). Given the different subunits and substrates of the two mTOR complexes, it is increasingly recognized that these complexes are distinct in their physiological roles and have different consequences to their activation and dysregulation in cancer (Morrison Joly *et al.*, 2016). RICTOR (rapamycin‐insensitive companion of mTOR) has been found to be overexpressed in various cancers (e.g., gastric and lung) and to be capable of cooperating with other driver mutations to stimulate cellular proliferation (Cheng *et al.*, 2015; Kim *et al.*, 2016; Morrison Joly *et al.*, 2016). Furthermore, in both glioblastoma and breast cancer, RICTOR/mTORC2 has been implicated as a mediator of disease progression and therapy resistance (Masri *et al.*, 2007; Morrison Joly *et al.*, 2016).

While relatively little is known about the intricacies of mTORC2 signaling in HNSCC, there is substantial interest in targeting the PI3K pathway (e.g., via targeted inhibition of PI3K, Akt, or mTORC1). Presently however, patient responses to PI3K inhibition have been variable and relatively short‐lived (Jimeno *et al.*, 2015; Juric *et al.*, 2018; Rodon *et al.*, 2014). Here, we have explored RICTOR/mTORC2 in HNSCC tumor cells and interrogated the role of mTORC2 in modulating response to PI3K inhibition. As targeted inhibition of mTORC2 has been explored infrequently to date, we have generated a novel gene knockout model of RICTOR using CRISPR/Cas9 to determine how loss of RICTOR/mTORC2 activity affects the therapeutic response of HNSCC tumor cells to PI3K inhibition, as well as to EGFR inhibition and cisplatin treatment.

## 2 Materials and methods

### 2.1 Tissue microarray and immunohistochemistry

Study approval was obtained from the University of Western Ontario Research Ethics Board (HSREB 103886). A retrospective search of the London Health Sciences Centre pathology database was performed to identify pretreatment oropharyngeal cancer biopsy specimens, and clinicopathological factors were extracted through a retrospective chart review. A tissue microarray (TMA) was constructed from 1‐mm core punches of primary site biopsy specimens; 4‐µm sections were cut and tested for RICTOR expression using immunohistochemistry (IHC) (ab70374; 2 µg·mL^−1^). RICTOR expression was scored by two clinical pathologists (PP and CJH) based on a combination of staining intensity [none (0), weak (1), moderate (2), strong (3)] and extent of staining (incomplete, complete). Disagreements in scoring were resolved by consensus. Quantitative reverse transcription PCR (qRT‐PCR) was used to test for HPV types 16 and 18 in DNA extracted from formalin fixed samples as previously described (Nichols *et al.*, 2013). RICTOR expression was compared to clinicopathologic variables by using Fisher’s exact tests and chi‐square tests. Survival curves stratified by RICTOR expression were generated and compared using log‐rank tests.

### 2.2 Immunoblotting and co‐immunoprecipitation

Cell lysates were prepared and analyzed by immunoblotting as described previously (Ruicci *et al.*, 2018b). A list of primary antibodies is provided in Table S1.

For co‐immunoprecipitation (co‐IP), cells were cultured in 15‐cm dishes, then washed with cold 1× PBS, pelleted, and re‐suspended in buffer (50 mm Tris/Cl, pH 7.5, 100 mm NaCl, 5% glycerol). Cells were again pelleted and lysed in 150–300 µL of buffer containing: 50 mm Tris/Cl pH 7.5, 100 nm NaCl, 5% glycerol, 1% NP‐40, 5 mm NaF, 1 mm PMSF, 1 mm DTT, and 1% Protease/Phosphatase inhibitors (Sigma‐Aldrich, St. Louis, MO, USA) for 15 min on ice. Cells were pelleted, and the protein content of the supernatant was determined by Bradford Assay. About 800 µg to 1 mg protein in 150–200 µL lysis buffer was used for co‐IP analysis. mTOR primary antibody (Table S1) was added to samples at 1 : 100. Samples were incubated, rotating, overnight at 4 °C. Protein G Dynabeads™ (10003D, Invitrogen, Carlsbard, CA, USA) were then added and incubated 2 h of rotating at 4 °C. Adhered protein complexes were collected using a magnet and washed several times by moving the beads through lysis buffer. 5× SDS was added and incubated 10 min at 70 °C to disrupt binding between the beads and proteins. Proteins were then analyzed by immunoblotting, as described. Membranes were visualized following exposure to enhanced chemiluminescence reagent (Luminata™ Crescendo or Luminata™ Forte, Western HRP Substrate; Millipore, Burlington, MA, USA) on a Bio‐Rad ChemiDoc™MP Imaging System (Hercules, CA, USA).

### 2.3 Quantitative real‐time PCR

Total RNA was extracted using AllPrep DNA/RNA Mini Kits (Qiagen). Eluted RNA was reverse transcribed to complementary DNA (cDNA) using QuantiTect Reverse Transcription Kits (Qiagen, Hilden, Germany). qRT‐PCR was then performed in 20‐µL reactions, using 2× Power SYBR® Green PCR Master Mix (Thermo Fisher Scientific, Waltham, MA, USA), 200 nm each of forward and reverse primers and 100 ng cDNA. PCR conditions: 95 °C for 10 min, followed by 45 cycles of 95 °C for 10 min, 95 °C for 15 s, 59 °C for 1 min, 72 °C for 40 s, with a melt curve: 95 °C for 10 s, 65 °C for 5 s, 95 °C for 50 s. Relative transcript abundance was determined using the delta‐delta CT method with expression of human β‐actin used for normalization. Primers (5′–3′): RICTOR (F‐AGTACGAGGGCGGAATGACA, R‐TGATACTCCCTGCAATCTGGC) and β‐actin (F‐AGAGCTACGAGCTGCCTGAC, R‐AGCACTGTGTTGGCGTACAG).

### 2.4 RICTOR overexpression studies

Plasmid DNA for a construct containing myc‐tagged RICTOR (11367, Addgene, Watertown, MA, USA) was prepared by mini‐prep (QIAprep^®^ Spin Miniprep Kit; Qiagen). For transfection, cells were plated into 6‐well dishes (300 000 cells/well) in antibiotic‐free media and allowed to attach overnight. The next day, 5 µg plasmid DNA was delivered in 5 µL P3000 reagent in Opti‐MEM^®^ with 3.75 µL Lipofectamine™ 3000 reagent (Thermo Fisher Scientific) in Opti‐MEM^®^, following a 15‐min incubation at room temperature. The next day, new antibiotic‐free media was added and cells were allowed to recover for 1–2 days. Cells were then collected or re‐plated for downstream assays. Overexpression of RICTOR was confirmed by immunoblotting.

### 2.5 CRISPR/CAS9‐mediated deletion of RICTOR

As no mTORC2‐specific inhibitors exist to date, CRISPR/Cas9 was used to delete a region of the *RICTOR* gene sequence, with the goal of diminishing the activity of mTORC2. A 132 base pair (bp) region encompassing exon 5 of the *RICTOR* gene was selected for targeted deletion (Fig. S2a). Two single‐guide (sg)RNA oligo sequences were designed (one upstream and one downstream of exon 5). Complimentary oligos were ordered for each guide sequence, and annealed guides were ligated into pSpCas9(BB)‐2A‐GFP (Addgene; 48138)‐CMV vectors (PX458‐CMV). Plasmid DNA was prepared using a QIAprep^®^ Spin Miniprep Kit (Qiagen), and ligations were verified by Sanger Sequencing (London Regional Genomics Centre). FaDu and Cal27 HNSCC cells were seeded in 24‐well dishes (50 000 cells/well), and 24 h later, 1 µg total plasmid DNA (500 ng each of the upstream and downstream guides) was delivered using Lipofectamine 3000 Reagent (Thermo Fisher Scientific) in Opti‐MEM^®^ (FaDu) or using FuGENE^®^ HD Transfection Reagent (Promega Corporation, Madison, WI, USA) (Cal27). Twenty‐four hours later, media was replaced and cells were allowed to recover for 24 h. PCR with Phusion^®^ High‐Fidelity DNA Polymerase was then used to genotype *RICTOR* exon 5; 20‐µL reactions were prepared, containing 5× Phusion GC Buffer, 0.4 µL of 10 mm dNTPs, 0.5 µL of 20 µm forward and reverse primers, and 0.2 µL Phusion. PCR conditions: 98 °C for 30 s, followed by 40 cycles of 98 °C for 10 s, 58 °C for 10 s, 72 °C for 20 s, then 72 °C for 5 min. PCR amplicons were run on 2% agarose gels, and the detection of a ~ 100 bp difference in product size was used to assess the presence of a deletion. Limiting dilutions were then used to deliver 1 cell/well into 96‐well plates. Following dilution, plates were incubated at 37 °C and media was changed as needed. Colonies were first visible after ~ 1 week. Once single cell colonies covered > 50% of the well, a pipette tip was used to wipe through the monolayer. Collected cells were deposited into PCR tubes and used to genotype *RICTOR* exon 5 (as described). Colonies with putative deletions were expanded for downstream assays and Sanger Sequencing) to determine the exact deletion regions. Primers (5′–3′) for *RICTOR* (F‐TTGAAACCTGTGCAGCAAAA, R‐CGTCCAACACACAATGCTCA).

### 2.6 Clonogenic survival assays

Cells were seeded at 500 cells/well into 24‐well dishes. Cells were allowed to adhere for 48 h at which time half of the wells for each cell line were treated with media containing 5 µm of the PI3K inhibitor BYL719 (alpelisib), 1 µm cisplatin, or 2.5 µm of the EGFR inhibitor erlotinib. For the next ~ 10 days, cells were monitored and drug‐containing media replaced as needed until visible colonies formed. Plates were then rinsed with 1× PBS, fixed with cold 100% methanol (MeOH), and stained with 0.5% crystal in 25% MeOH/1× PBS. Plates were washed with water and air‐dried. Number of colonies was quantified using the fiji plugin for ImageJ (NIH, Bethesda, MD, USA).

### 2.7 Cell viability assays

Cells were seeded in 96‐well plates in drug‐free media at 2400 cells/well. 24 h later, media was removed and replaced with drug‐containing media over a 10‐point dose range for each drug 0–40 µm. Cells were incubated for 72 h at 37 °C in 95% air and 5% CO_2_. Cell viability was then measured indirectly using the PrestoBlue^®^ Cell Viability Reagent (Thermo Fisher Scientific), following a 1‐h incubation at 37 °C using a Synergy™ H4 Hybrid Reader (BioTek, Winooski, VT, USA) with 560 nm excitation and 590 nm emission wavelengths. For each dose, viability values were normalized to no‐drug controls and average viability for each dose was calculated.

### 2.8 Statistical analysis

All analyses were performed with prism ^®^ 7 graphpad Software (GraphPad, San Diego, CA, USA). Experimental groups were compared with controls using Student’s unpaired, two‐tailed *t‐*tests. Multiple groups were compared across a single condition using one‐way ANOVA. Significance of clinicopathological features was assessed as described. *P* < 0.05 was used to define significant differences from the null hypothesis.

## 3 Results

### 3.1 RICTOR/mTORC2 is overexpressed in a subset of HNSCC primary tumors

We began by examining the prevalence of genomic aberrations and altered RNA expression of mTORC2 components in HNSCC patient tumors (Fig. 1B). While *DEPTOR* was found to be most frequently amplified, it is not unique to mTORC2 (Huang and Fingar, 2014; Sarbassov *et al.*, 2004). *RICTOR*, which is only found in mTORC2, was also amplified in a subset of cases (5.2%, *n* = 496 total cases examined) and overexpressed in others (62/496, 12.5%). *RICTOR* has been found to be overexpressed and/or amplified in various other cancers and to cooperate with other driver mutations to stimulate cellular proliferation (Cheng *et al.*, 2015; Kim *et al.*, 2016; Morrison Joly *et al.*, 2016). Because we were interested in the effect of mTORC2 signaling on the therapeutic efficacy of PI3K inhibition in HNSCC tumor cells, we made use of the cBioPortal interface to evaluate whether aberrations in these genes (*RICTOR* and *PIK3CA*) tend to co‐occur or be exclusive from one another. We found *PIK3CA* and *RICTOR* aberrations to significantly co‐occur in HNSCC tumors (Fig. 1C). In addition, we noted aberrations in *RICTOR* to also significantly co‐occur with those in *EGFR*, which is also frequently altered in HNSCC tumors and functions at the cell surface to transduce signaling to oncogenic pathways, including the PI3K pathway (Fig. 1C) (Lawrence *et al.*, 2015; Rodon *et al.*, 2013). The co‐occurrence of *RICTOR* aberrations with prominent HNSCC driver alterations is important, as RICTOR overexpression is associated with increased mTORC2 activity (Laugier *et al.*, 2015). If patients with *PIK3CA‐* and *EGFR*‐altered HNSCC tumors are to be candidates for either PI3K or EGFR inhibitors, it may be a relevant therapeutic consideration that a subset of these tumors also have alterations in, or show overexpression of RICTOR and therefore have mTORC2 activity that may continue to drive Akt activation (Janku *et al.*, 2011; Laugier *et al.*, 2015). Although RICTOR aberrations did not affect survival in the entire TCGA cohort, in both *PIK3CA* amplified and *EGFR* amplified HNSCC tumor subsets, there is a trend toward a shorter time to relapse in cases with *RICTOR* aberrations versus in those without (Fig. 1D).

### 3.2 Relation between RICTOR expression, clinicopathological variables, and survival

**Figure 2:**
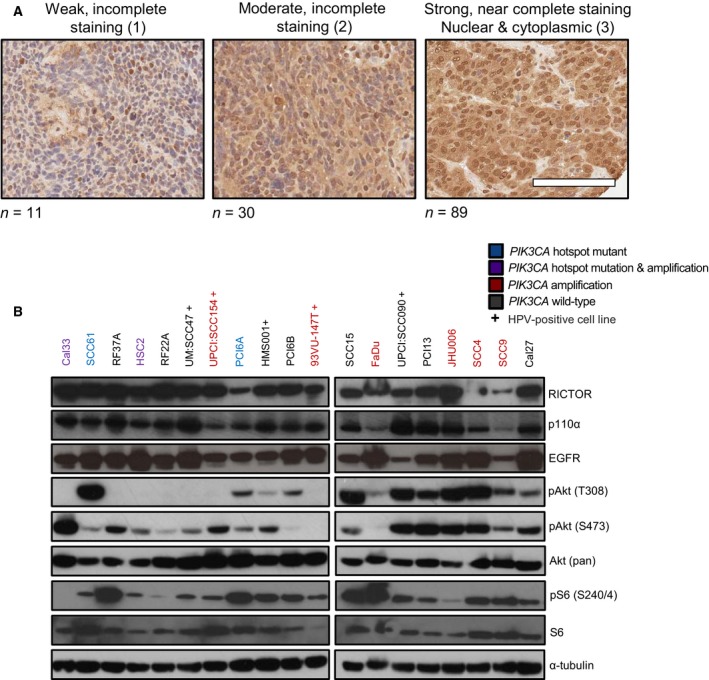
RICTOR/mTORC2 and PI3K pathway activation in established HNSCC cell lines and primary tumors. (A) Representative images of RICTOR IHC in human HNSCCs arranged by score. Scale bar represents 100 µm. (B) Immunoblot of RICTOR, EGFR, p110α, and Akt with lysates from indicated HNSCC cell lines. ‘+’ denotes HPV‐positive cell lines.

We used IHC to assess the expression of mTORC2 subunit RICTOR in clinical HNSCC TMAs composed of 130 HNSCC patients from our institution. RICTOR was expressed variably across the tumor tissues surveyed, with the majority of samples having strong and complete expression of RICTOR (score of 3), consistent with other studies that have found positive RICTOR IHC staining in the majority of HNSCC cases examined (Fig. 2A; Naruse *et al.*, 2017). RICTOR was detected in both the cytoplasm and the nucleus in some cases (Rosner and Hengstschlager, 2008). Eleven patients were scored as having weak, incomplete staining (8.5%; score = 1), 30 were scored as moderate, incomplete staining (23.1%; score = 2), and 89 were scored as strong, complete staining (68.5%; score = 3). No samples were assigned a score of 0. Scores 0/1 and 2/3 were grouped together for analyses *a priori*. Based on chi‐squared and Fisher’s exact tests, we found RICTOR expression positively related to tumor site (*P* = 0.0018) (Table S2). Analysis for survival (disease‐free and overall) revealed no differences between RICTOR expression groups (Fig. S1a,b).

### 3.3 Activated Akt and RICTOR are elevated in HNSCC cell lines

Nineteen HNSCC cell lines (including 5 HPV‐positive lines, denoted ‘+’) were profiled for the expression of RICTOR and other PI3K pathway members (Fig. 2B). RICTOR, p110α (encoded by *PIK3CA*), and EGFR were readily detected in all lines surveyed. In general, the expression pattern for RICTOR, EGFR, and p110α followed the same pattern between cell lines. Akt phosphorylated at Ser473 was detected in most cell lines at baseline, with fewer cells displaying Akt phosphorylated at Thr308.

### 3.4 Feedback relief following PI3K inhibition leads to Akt Ser473 accumulation

**Figure 3:**
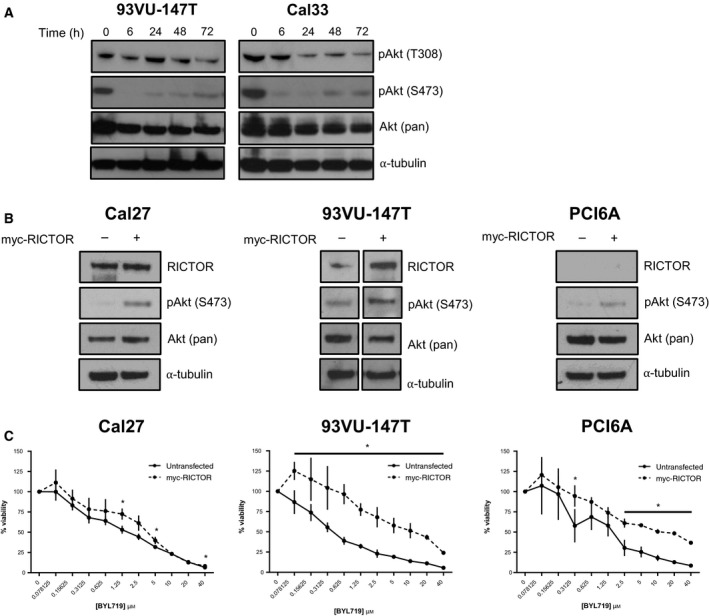
Feedback relief following PI3K inhibition leads to Akt Ser473 accumulation. (A) Immunoblot showing time‐dependent re‐accumulation of phosphorylated Akt (Ser473) following PI3K inhibition by BYL719 (5 µm). (B) Immunoblot with indicated antibodies following transfection of HNSCC cells with myc‐tagged RICTOR. (C) Proliferation after 72 h of HNSCC cell lines at baseline compared to following transfection of myc‐tagged RICTOR upon increasing doses of BYL719 (0–40 µm). *P < 0.05.

Inhibition of PI3K signaling is known to relieve negative feedback from S6K to RICTOR (Thr1135) (schematic shown in Fig. 1A). To evaluate how loss of negative feedback to RICTOR/mTORC2 affected Akt phosphorylation in HNSCC tumor cells, we examined Akt phosphorylation following PI3K inhibition by BYL719 (5 µm) for up to 72 h (Fig. 3A). While Akt Thr308 phosphorylation was variably suppressed, Akt Ser473 phosphorylation was steadily restored over time, as expected, based on the relief of S6K‐RICTOR negative feedback (Liu *et al.*, 2015).

### 3.5 RICTOR overexpression promotes resistance to PI3K inhibition

RICTOR overexpression is associated with elevated activity of the mTORC2 complex (Laugier *et al.*, 2015). To evaluate the effect of elevated mTORC2 activity on the response of HNSCC cells to PI3K inhibition, myc‐tagged RICTOR was exogenously expressed in Cal27, 93VU‐147T, and PCI6A HNSCC cells (Fig. 3B). When cell viability was measured following treatment with BYL719, cells overexpressing RICTOR showed increased viability for all three cell lines, indicative of a reduced response to PI3K inhibition relative to the control cells (Fig. 3C).

### 3.6 Generation of RICTOR knockout cells using CRISPR/Cas9

**Figure 4:**
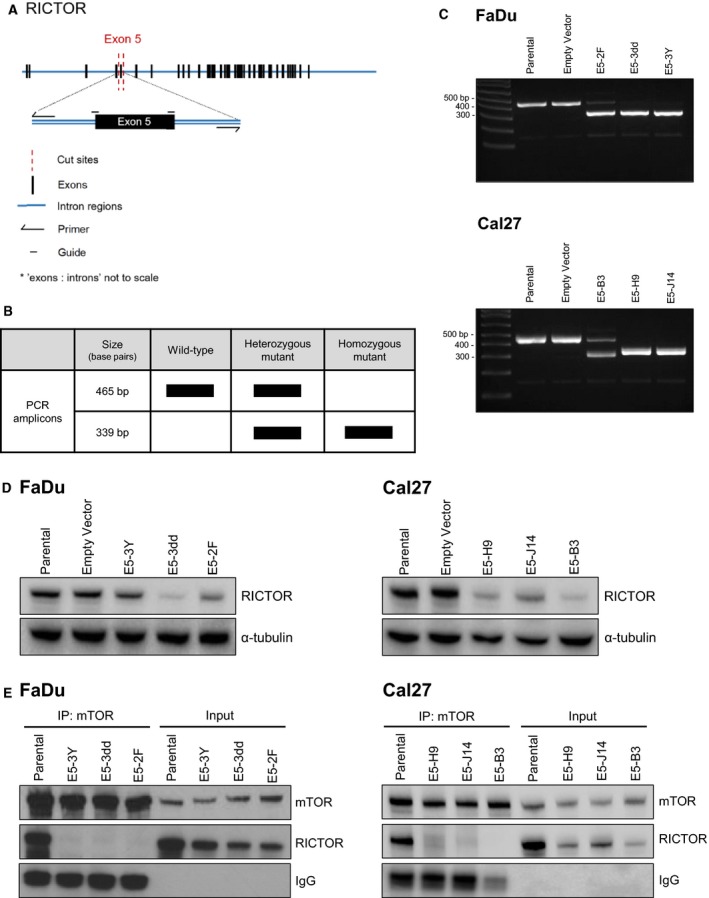
Deletion of *RICTOR* exon 5 disrupts the interaction between RICTOR and mTOR. (A) Schematic illustrating design of single‐guide RNA and primers for CRISPR/Cas9‐mediated deletion of exon 5 of *RICTOR.* (B) Predicted genotypes and base pair sizes for genomic PCR amplicons of *RICTOR* following CRISPR/Cas9 targeting of *RICTOR*. (C) Agarose gel images showing *RICTOR* amplicons in cell populations (FaDu, Cal27 cells) transfected with guides targeting *RICTOR* or an empty vector (PX458‐CMV). (D) Immunoblot of RICTOR expression in parental and mutant cell lines (E5‐XX lines). (E) Immunoblot showing co‐IP of RICTOR and mTOR in FaDu and Cal27 cells, but no detectable interaction in any of the putative *RICTOR* knockout cell lines. [Correction added on 06 January 2020, after first online publication: Fig. 4 has been amended. In the original publication of this article, the RICTOR immunoblot in Fig. 4D was accidently removed.]

Although numerous mTORC1‐selective and dual mTORC1/2 inhibitors exist, there has only been one report of a possible mTORC2 inhibitor, discovered through a high‐throughput yeast two‐hybrid screen (Benavides‐Serrato *et al.*, 2017; Rodon *et al.*, 2013). The efficacy of mTORC2‐specific inhibition in the context of HNSCC therefore remains unknown. We addressed this by generating a genetic knockout of RICTOR using CRISPR/Cas9 technology. Specifically, we targeted exon 5 of *RICTOR* using two custom single‐guide RNA (Fig. 4A, additional detail in Fig. S2). The protein domain structure of RICTOR and mTORC2 has only recently been elucidated in humans; as exon 5 (residues 88–130) occurs near the beginning of the large *RICTOR* gene and is contained within the N‐terminal armadillo (ARM) repeat cluster that is known to interact with mTOR, we hypothesized that disrupting this region may impair mTORC2 activity (Fig. S3; Chen *et al.*, 2018; Zhou *et al.*, 2015).

The predicted deletion products for our CRISPR/Cas9 model are shown in Fig. 4B. *RICTOR* deletion was attempted in two HNSCC cell lines: Cal27 and FaDu. Both cell lines are among the least sensitive to PI3K inhibition (Fig. 2A), and FaDu contains a *PIK3CA* amplification (Fig. 2B; Ruicci *et al.*, 2018a). As we noted *PIK3CA*‐amplified TCGA tumors that also harbored *RICTOR* aberrations tended to relapse more quickly, testing the effect of mTORC2 loss in this setting was of interest. Following the transfection of the single‐guide (sg)RNA into FaDu and Cal27 cells, single‐cell clones were generated by limiting dilutions and expanded before verifying deletion of exon 5 by PCR. Three clonal cell lines are shown per parental line (Fig. 4C). We used Sanger sequencing to validate the deletion of exon 5 in each cell line. Sequences were aligned to the wild‐type *RICTOR* gene sequence, confirming the presence of the predicted deletion (Fig. S5). As a control, we also amplified and sequenced the top 2 off‐target putative binding sites for both guide RNA used; no mutations were detected.

We proceeded to evaluate RICTOR expression by immunoblotting in each putative knockout line. Immunoblotting revealed that in all knockout lines, RICTOR protein was still detectable at the correct size (200 kDa; Fig. 4D) with no apparent truncated protein versions visible at smaller sizes (Fig. S4). This was true for knockout cell lines with identical exon 5 (E5) deletions in both alleles (FaDu E5‐3Y and E5‐3dd, Cal27 E5‐J14 and E5‐H9) and for RICTOR knockout cell lines with the predicted deletion in 1 allele and a smaller deletion in the other allele (FaDu E5‐2F and Cal27 E5‐B3). In some cell lines (e.g., E5‐B3, E5‐H9, and E5‐3dd), RICTOR protein appeared to be in lower abundance than in parental lines; however, in all cell lines, a 200 kDa band was visible. Of note, the RICTOR antibody used targets a region coded within exon 31 (leucine residue 1121 specifically) of RICTOR. It is conceivable that, even with deletion of the 42‐codon exon 5 (residues 88–130), an altered protein may still form. Relative to the total size of RICTOR, which contains 1732 codons, loss of 42 codons would result in a size difference of just 4.62 kDa.

### 3.7 RICTOR exon 5 deletion disrupts RICTOR/mTOR binding

We next sought to evaluate whether the biological activity of RICTOR in the knockout cell lines was still intact. We used co‐IP to evaluate binding between mTOR and RICTOR. While mTOR/RICTOR binding was readily detected in parental FaDu and Cal27 cells, no apparent interaction between mTOR and RICTOR was detected in any of the knockout lines tested (Fig. 4E). This observation suggests that loss of *RICTOR* exon 5 impairs the interaction between RICTOR and mTOR that is necessary to produce the mTORC2 complex.

### 3.8 RICTOR/mTORC2 loss reduces colony forming ability and cell line growth

**Figure 5:**
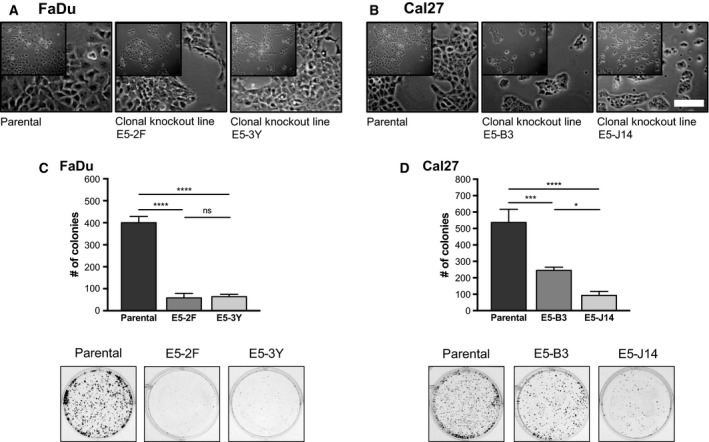
Deletion of *RICTOR* exon 5 alters cell growth and colony forming ability. Phase contrast images of parental and *RICTOR* knockout FaDu (A) and Cal27 (B) cell lines. Scale bar represents 130 µm. (C, D) Colony formation assays of parental and *RICTOR* knockout cell lines following 10 days of growth. Number of colonies was quantified using fiji software. Error bars represent SD, *n* = 3. *P < 0.05, ***P < 0.001, ****P < 0.0001, ns, not significant, one‐way ANOVA.

Having confirmed the absence of mTORC2 formation in RICTOR knockout cells, we proceeded to characterize the clonal knockout cell lines generated from both FaDu and Cal27 parental cells. Morphologically, FaDu RICTOR knockout cells and parental cells appeared similar (Fig. 5A), whereas Cal27 RICTOR knockout cells tended to grow in smaller, dense colonies, rather than in ‘sheets’ as seen for the parental cells (Fig. 5B). In terms of colony forming ability, both FaDu and Cal27 RICTOR knockout lines were drastically impaired when challenged to grow as single colonies, with significantly fewer colonies present in RICTOR knockout Cal27 and FaDu cells relative to parental cells (Fig. 5C,D).

### 3.9 Activating phosphorylation of Akt is lost in RICTOR knockout cell lines

**Figure 6:**
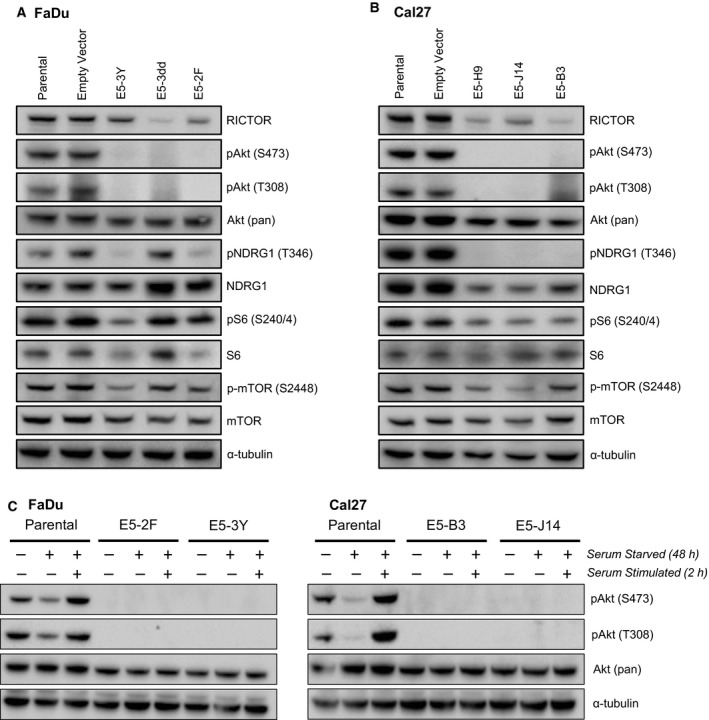
Activating phosphorylation of Akt is lost in RICTOR knockout cell lines. (A, B) Immunoblots of indicated lysates showing activation status of mTORC2 readouts Akt (Ser473) and NDRG1 (Thr346), as well as expression of other relevant pathway members. (C) Immunoblots of indicated lysates showing phosphorylation of Akt (Thr308 and Ser473) following serum starvation for 48 h and serum stimulation for 2 h.

We next examined the status of mTORC2 readouts by immunoblotting. No phosphorylated Akt was detected in any knockout line – neither Akt Ser473, nor Akt Thr308 (Fig. 6A,B), although there was no loss of endogenous Akt. The temporal sequence of Akt phosphorylation is somewhat debated in the literature, but the predominant view is that Akt Ser473 phosphorylation typically precedes Thr308 phosphorylation and that its presence boosts the subsequent phosphorylation of Thr308 (Sarbassov *et al.*, 2004). Based on our results, it appears that the lack of Akt Ser473 phosphorylation (due to impaired mTORC2 activity) impairs Akt Thr308 phosphorylation to the extent that it is absent or nearly undetectable (Sarbassov *et al.*, 2004; Scheid *et al.*, 2002). Using the HNSCC cohort (*n* = 346) from The Cancer Proteome Atlas (TCPA), it is apparent that phosphorylation of Akt Ser473 and Akt Thr308 is tightly correlated (*P* = 7.7526 × 10^−19^) in HNSCC tumors (Fig. S6a), although increased or reduced Akt phosphorylation does not appear to correlate with the presence or absence of *RICTOR* aberrations (Fig. S6b). To verify that the Akt Thr308 kinase PDK1 was still present in RICTOR knockout cells, we evaluated PDK1 expression by immunoblotting and detected it in all lines (Fig. S6c). Apart from Akt, we also examined phosphorylation of NDRG1. NDRG1 is an established readout for activity of serum/glucocorticoid regulated kinase 1 (SGK1), which is a direct phosphorylation target of mTORC2 (Castel *et al.*, 2016). SGK1 has been reported to be difficult to detect reliably by western blot; therefore, its substrate NDRG1 is typically surveyed (Castel *et al.*, 2016). Phosphorylation of NDRG1 was lost in most RICTOR knockout clones, with the exception of E5‐3dd. Phosphorylation of the ribosomal protein S6 was also variably reduced across the knockout cell lines, as was phosphorylation of mTORC1 (Ser2448; active form) (Fig. 6A,B).

While Akt Ser473 and Thr308 phosphorylation was absent at baseline in all RICTOR knockout lines, we proceeded to determine whether, under a condition of cellular stress, activated Akt could be detected. We conducted a serum starvation assay in which each cell line was starved for 48 h and then serum‐stimulated for 2 h. In parental FaDu and Cal27 cells, starvation reduced Akt phosphorylation at both sites, which was then rescued by serum stimulation (Fig. 6C). In contrast, in all knockout lines analyzed, serum starvation followed by stimulation was unable to induce any level of Akt phosphorylation at either phosphorylation site, suggesting total impairment of mTORC2 in phosphorylating Akt Ser473 and confirming the necessity of Ser473 phosphorylation for subsequent phosphorylation of Akt at Thr308.

### 3.10 RICTOR/mTORC2 loss sensitizes HNSCC cells to PI3K inhibition

**Figure 7:**
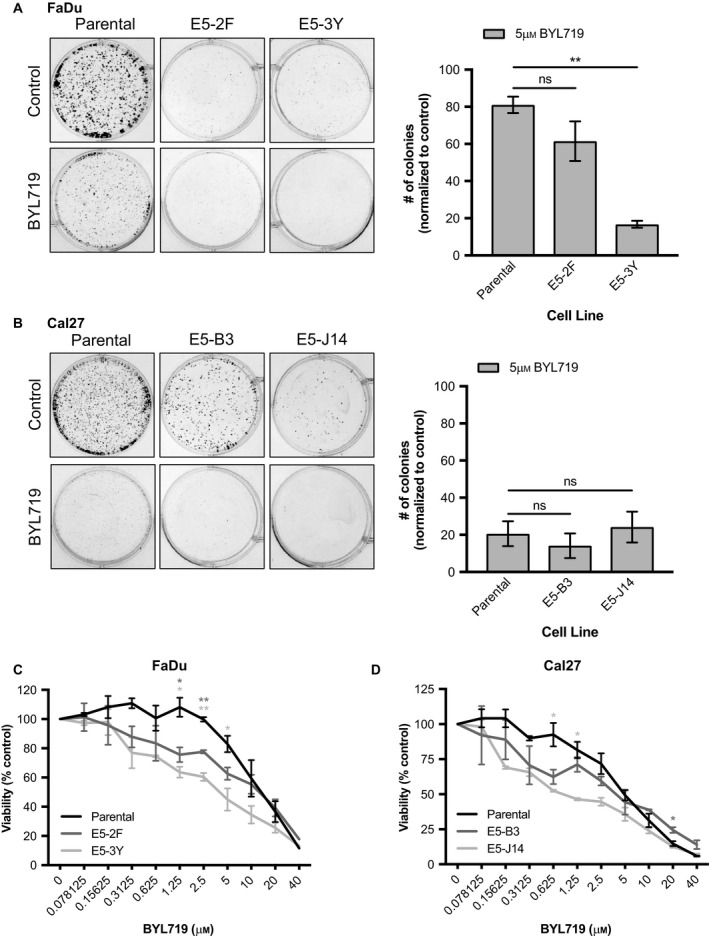
RICTOR/mTORC2 loss improves response of HNSCC cells to PI3K inhibition. (A, B) Colony formation assays of parental and *RICTOR* knockout cell lines with/without 5 µm BYL719 for 10 days. Number of colonies was quantified using fiji software. Error bars represent SD. *n* = 3. (C, D) Proliferation after 72 h of parental versus RICTOR/mTORC2 knockout HNSCC cells upon increasing doses of BYL719 (0–40 µm). Error bars represent SEM. *P < 0.05, **P < 0.01, ns, not significant, one‐way ANOVA.

Due to the recovery of mTORC2 activity that occurs following PI3K/Akt/mTORC1 axis inhibition, we proceeded to examine whether co‐targeting mTORC2 alongside PI3K inhibition may enhance its anticancer efficacy. As mentioned, specific inhibitors of mTORC2 have not been investigated to date. We therefore made use of our RICTOR knockout cell lines and interrogated their relative responsiveness to PI3K inhibition. In the presence of the PI3K inhibitor BYL719, we found RICTOR knockout lines to be impaired in their ability to form colonies. Because RICTOR knockout cells showed poor colony forming ability even without drug treatment (Fig. 5C), we normalized the number of colonies following BYL719 treatment to the number of colonies present in corresponding untreated wells for each clonal line (Fig. 7A,B). In FaDu cells, the cell line E5‐3Y (which has complete *RICTOR* exon 5 loss in both alleles) showed a significantly enhanced response to BYL719 (*P* < 0.01). In terms of sensitivity to BYL719, we evaluated cellular viability of parental and RICTOR knockout cells in response to BYL719 across a 10‐point dose range. For both FaDu and Cal27, all RICTOR knockout cell lines showed increased sensitivity to BYL719 (Fig. 7C,D).

### 3.11 RICTOR/mTORC2 loss improves response of HNSCC cells to erlotinib and cisplatin

**Figure 8:**
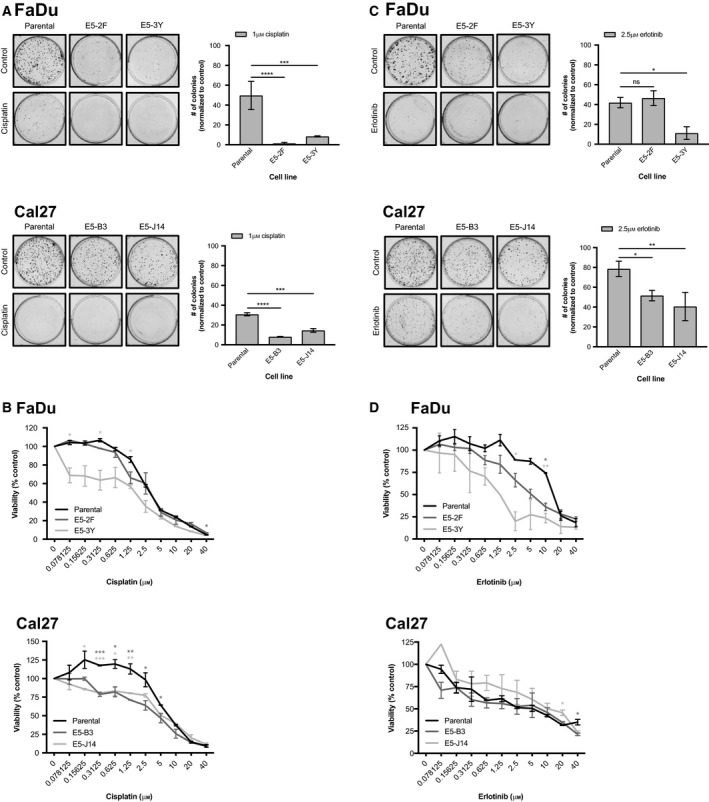
RICTOR/mTORC2 loss sensitizes HNSCC cells to erlotinib and cisplatin treatment. (A) Colony formation assays of parental and *RICTOR* knockout cell lines with/without 1 µm cisplatin for 10 days. Number of colonies was quantified using fiji software. Error bars represent SD. *n* = 3. (B) Proliferation after 72 h of parental versus RICTOR/mTORC2 knockout HNSCC cells upon increasing doses of cisplatin (0–40 µm). Error bars represent SEM. (C) Colony formation assays of parental and *RICTOR* knockout cell lines with/without 2.5 µm erlotinib for 10 days. Number of colonies was quantified using fiji software. Error bars represent SD. *n* = 3. (D) Proliferation after 72 h of parental versus RICTOR/mTORC2 knockout HNSCC cells upon increasing doses of erlotinib (0–40 µm). Error bars represent SEM. *P < 0.05, **P < 0.01, ***P < 0.001, ****P < 0.0001, ns, not significant, one‐way ANOVA.

To extend the applicability of mTORC2 as a target in HNSCC, we lastly examined the efficacy of the EGFR inhibitor erlotinib and the alkylating chemotherapy agent cisplatin in cells lacking mTORC2 activity. EGFR is an established therapeutic target in HNSCC as it is known to be frequently amplified (Lawrence *et al.*, 2015). Cisplatin is one of the most frequently used chemotherapies for HNSCC patients, and mTORC2 has previously emerged as a mediator of resistance to cisplatin therapy in ovarian cancer (Im‐aram *et al.*, 2013). In both FaDu and Cal27 cells, RICTOR knockout resulted in significantly fewer colonies able to grow in the presence of cisplatin (Fig. 8A). In Cal27, both RICTOR knockout cell lines tested showed significantly reduced cellular viability when treated with cisplatin, relative to the parental line (Fig. 8B). In FaDu cells, E5‐3Y cells showed a significant reduction in viability following cisplatin treatment.

In response to erlotinib, FaDu cells lacking mTORC2 activity showed reduced cellular viability and significantly impaired colony forming assay relative to the parental cell line (Fig. 8C,D). Cal27 cells lacking mTORC2 activity showed significant reductions in colony forming ability with erlotinib treatment; however, cell viability was not measurably reduced (Fig. 8C,D).

## 4 Discussion

mTOR integrates oncogenic PI3K/Akt signaling and downstream pathways regulating cell growth, metabolism, survival, and protein synthesis (Engelman, 2009). mTOR itself exists in two structurally and functionally distinct multiprotein complexes – mTORC1 and mTORC2 (Sarbassov *et al.*, 2004). Whereas mTORC1 is activated downstream of PI3K/Akt, the lesser‐known mTORC2 functions upstream of Akt (Sarbassov *et al.*, 2004). Inhibition of PI3K/Akt/mTORC1 is known to cause a recovery of mTORC2 activity, due to loss of feedback inhibition (Huang and Fingar, 2014; Sarbassov *et al.*, 2004). As such, it is thought that re‐activation of mTORC2 may play a role in dampening the therapeutic effect of PI3K inhibition and may therefore serve as an important oncogenic inhibitory target.

The studies presented here examine RICTOR/mTORC2 signaling in HNSCC specifically, where PI3K inhibition is one of the leading targeted therapies currently under investigation (Lui *et al.*, 2013; Rodon *et al.*, 2013). To date, efficacy of PI3K inhibitors, both preclinically and in clinical trials, has been variable; recovery of mTORC2‐mediated Akt activation following PI3K inhibition may be a central adaptive resistance mechanism limiting their efficacy (Gkountakos *et al.*, 2018; Huang and Fingar, 2014; Sarbassov *et al.*, 2004). In both HNSCC primary tumors and a panel of HNSCC cell lines, we detected abundant RICTOR expression, the defining subunit of mTORC2. Elevated expression of RICTOR is known to promote increased mTORC2 activity, and RICTOR is essential for the catalytic activity of mTOR (Laugier *et al.*, 2015). Previous studies have similarly found oral squamous cell carcinomas to exhibit positive RICTOR IHC staining in the majority of cases surveyed (68%) (Naruse *et al.*, 2017). We observed genomic aberrations in *RICTOR* and *PIK3CA* to significantly co‐occur in HNSCC tumors; as patients with *PIK3CA* aberrations are thought to be optimal candidates for PI3K inhibition therapy, the prevalence of *RICTOR* amplifications or RNA overexpression in this cohort may have therapeutic implications (Gkountakos *et al.*, 2018). The possibility that *RICTOR* amplification may affect PI3K/Akt/mTORC1 inhibition is already under evaluation in three phase II clinical trials with the dual mTORC1/2 inhibitor AZD2014 (NCT03106155, NCT03166904, NCT03061708) (Gkountakos *et al.*, 2018). In these trials, *RICTOR* is being assessed for amplification and/or for protein overexpression by IHC (Gkountakos *et al.*, 2018).

Further support for RICTOR/mTORC2 activity modulating response to PI3K inhibition is provided by our observation that across HNSCC cell lines, phosphorylated Akt (Thr308 and Ser473) was highest in cell lines that were less susceptible to PI3K inhibition. Akt Ser473 phosphorylation is mediated by mTORC2 directly, and Akt Thr308 phosphorylation, although not mediated by mTORC2, is thought to be ‘primed’ by phosphorylation of Ser473 (Sarbassov *et al.*, 2004). These observations collectively suggest that the activity of mTORC2 may influence the sensitivity of HNSCC tumor cells to PI3K inhibition.

Owing to the paucity of mTORC2‐specific inhibitors available presently, and the limited understanding of mTORC2 signaling relative to mTORC1 and other PI3K pathway effectors, we used CRISPR/Cas9 to delete a region of *RICTOR* and disrupt the function of mTORC2. Our goal was to then interrogate the impact of mTORC2 impairment both at baseline, and when combined with PI3K inhibition and other chemotherapies. Following deletion of *RICTOR* exon 5, the interaction between RICTOR and mTOR was abolished, despite the detection of substantial levels of RICTOR. This observation identifies the region encoded by exon 5 as essential for the interaction of RICTOR with mTOR and therefore essential to the formation of mTORC2 (Zhou *et al.*, 2015). As a specific interdependence between RICTOR and the SIN1 subunit of mTORC2 has been described, an examination of the changes in SIN1 expression and binding following RICTOR deletion in our cell lines may inform the specific roles of these subunits in relation to each other (Oh and Jacinto, 2014).

Consistent with impaired mTORC2 formation, RICTOR deletion eliminated all activating Akt Ser473 phosphorylation. Importantly, phosphorylation of Akt Thr308 was also absent in all RICTOR knockout lines. Our results therefore highlight the importance of Ser473 phosphorylation for subsequent Thr308 phosphorylation (Sarbassov *et al.*, 2004). Further, these observations lead us to speculate as to whether blockade of mTORC2 alone may be sufficient to substantially impair Akt activity and therefore serve as a key therapeutic target. The selection of mTORC2 as an anticancer target is additionally supported by the impaired cell growth and colony forming ability of RICTOR knockout cells.

Relative to parental cells, RICTOR/mTORC2 knockout cells showed, in most cases, significant improvements in responsiveness to PI3K inhibition, EGFR inhibition, and cisplatin treatment. Several recent preclinical studies have demonstrated mTORC2 inhibition to be critical for the efficacy of various targeted agents, including the HER2 inhibitor lapatinib and CDK4/6 inhibitors (Morrison Joly *et al.*, 2016; Zhang *et al.*, 2016). Our findings contribute to a growing body of evidence highlighting mTORC2 as a central signaling node and a promising target or co‐target in cancer.

## 5 Conclusions

To date, most studies addressing PI3K/Akt/mTOR signaling have focused on either PI3K inhibition, or on downstream mTORC1 inhibition (Engelman, 2009). While inhibitors of PI3K and mTORC1 have demonstrated therapeutic efficacy in certain contexts, a gradual re‐accumulation of Akt Ser473 phosphorylation is typically observed, leading to a restoration/re‐activation of PI3K signaling (Gkountakos *et al.*, 2018). Increasingly, mTOR kinase inhibitors targeting both mTORC1 and mTORC2 are being investigated, although specific mTORC2 inhibitors have not been thoroughly explored established (Benavides‐Serrato *et al.*, 2017; Huang *et al.*, 2013; Li *et al.*, 2013). If toxicity associated with dual mTORC1/2 inhibition is limiting, then specific inhibition of mTORC2 may be a promising therapeutic avenue, particularly given its ability to diminish activating Akt Ser473 and Thr308 phosphorylation alike (Sarbassov *et al.*, 2004).

In summary, our analyses of HNSCC patient tumors and cell lines combined with a novel CRISPR/Cas9‐mediated genetic knockout of RICTOR reveal a key oncogenic role for RICTOR/mTORC2 in HNSCC. We find RICTOR/mTORC2 blockade to impair cellular viability and growth and to enhance the efficacy of PI3K and EGFR inhibitors, as well as cisplatin. These observations support the ongoing push for the development of a specific mTORC2‐targeting agent for use in cancer treatment and for further investigations centered on understanding the regulation and cellular activities of mTORC2.

## Conflict of interest

The authors declare no conflict of interest.

## Author contributions

KMR and JWB completed all experiments, including design of CRISPR/Cas9 guides. SSD, WS, and CJH generated the tissue microarray used in this study. PP and CJH completed the tissue microarray IHC scoring. All authors were involved in study design and manuscript preparation.

## Supporting information


**Fig. S1.** (A) Overall survival of HNSCC cases (n = 130) from the London Health Sciences Centre (LHSC), stratified by RICTOR IHC score (scores 0 & 1, versus 2 & 3). Cases scored as having RICTOR expression of 2 or 3 (n = 119) are represented in red. (B) Disease‐free survival of HNSCC cases (n = 130), stratified by RICTOR IHC score (scores 0 & 1, versus 2 & 3). Cases scored as having RICTOR expression of 2 or 3 (n = 119) are represented in red.

**Fig. S2.** Schematic illustrating design of single‐guide RNAs and primers for CRISPR/Cas9‐mediated deletion of exon 5 of *RICTOR.*

**Fig. S3.** (A) Schematic representation of predicted domains of the human *RICTOR* gene. (B) Schematic representation of mTOR domains, with putative interacting HEAT domains of RICTOR and mTOR shown. Domain sizes not shown to scale. Adapted from Zhou P, *et al.*, J Comp Bio, 2015.

**Fig. S4.** Immunoblot of RICTOR expression in parental and putative RICTOR knockout cell lines (E5‐XX lines). Full‐length gel shown in order to evaluate the presence of any truncated proteins forming following RICTOR exon 5 deletion.

**Fig. S5.** Sequencing alignments for (A) FaDu and (B) Cal27 cell lines. RICTOR knockout cell lines underwent Sanger Sequencing and were aligned to their parental counterparts, revealing deletions of variable sizes in the *RICTOR* gene sequence spanning exon 5. The wild‐type gene sequence is at the top of each panel, indicated in bold text.

**Fig. S6.** (A) Correlation between abundance of Akt (Thr308) and Akt (Ser473) in HNSCC primary tumor samples curated by The Cancer Proteome Atlas (TCPA). (B) Correlation between abundance of Akt (Thr308) and Akt (Ser473) in HNSCC primary tumor samples curated by TCPA, in relation to the presence or absence of *RICTOR* aberrations, as determined by TCGA. (C) Immunoblot of PDK1 expression in parental and RICTOR knockout cell lines (E5‐XX lines).

**Table S1.** Antibodies used in this study.

**Table S2.** Clinical and pathological characteristics of 130 patients with HNSCC and association with RICTOR expression.
