# Erratum to: Disruption of the RICTOR/mTORC2 complex enhances the response of head and neck squamous cell carcinoma cells to PI3K inhibition

**DOI:** 10.1002/1878-0261.12604

**Published:** 2019-12-05

**Authors:** Kara M. Ruicci, Paul Plantinga, Nicole Pinto, Mohammed I. Khan, William Stecho, Sandeep S. Dhaliwal, John Yoo, Kevin Fung, Danielle MacNeil, Joe S. Mymryk, John W. Barrett, Christopher J. Howlett, Anthony C. Nichols

In the original publication of this article, the RICTOR immunoblot in Fig. 4D was accidently removed as an error during production. The correct version of Fig. 4 is shown below.

**Figure 4:**
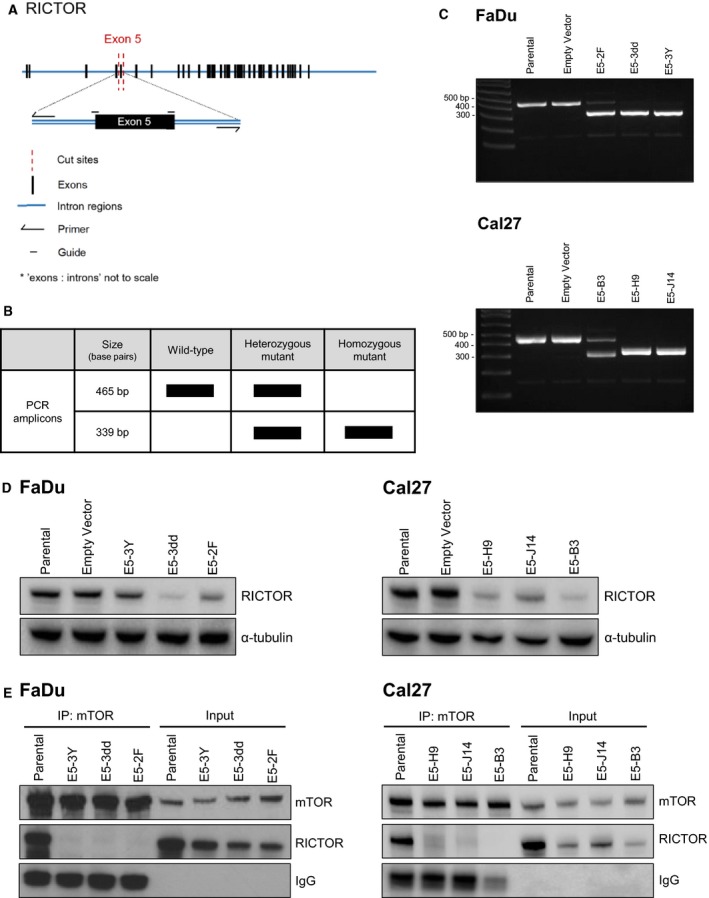
Deletion of *RICTOR* exon 5 disrupts the interaction between RICTOR and mTOR. (A) Schematic illustrating design of single‐guide RNA and primers for CRISPR/Cas9‐mediated deletion of exon 5 of *RICTOR.* (B) Predicted genotypes and base pair sizes for genomic PCR amplicons of *RICTOR* following CRISPR/Cas9 targeting of *RICTOR*. (C) Agarose gel images showing *RICTOR* amplicons in cell populations (FaDu, Cal27 cells) transfected with guides targeting *RICTOR* or an empty vector (PX458‐CMV). (D) Immunoblot of RICTOR expression in parental and mutant cell lines (E5‐XX lines). (E) Immunoblot showing co‐IP of RICTOR and mTOR in FaDu and Cal27 cells, but no detectable interaction in any of the putative *RICTOR* knockout cell lines.
