# Supplementary figures and images for: Disruption of the RICTOR/mTORC2 complex enhances the response of head and neck squamous cell carcinoma cells to PI3K inhibition
Source: Mol Oncol. 2019 Aug 28;13(10):2160–77. doi: 10.1002/1878-0261.12558 (PMC6763779; doi:10.1002/1878-0261.12558)

**A.**

### Overall Survival

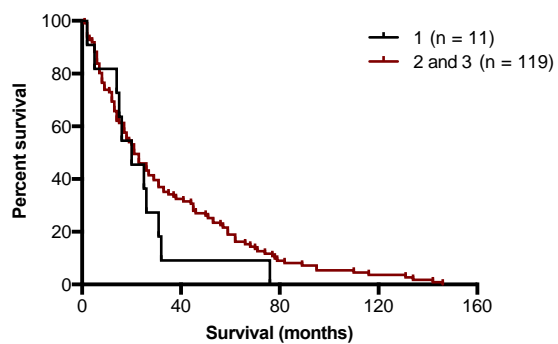

log-rank test  $p = 0.2783$

**B.**

### Disease-Free Survival

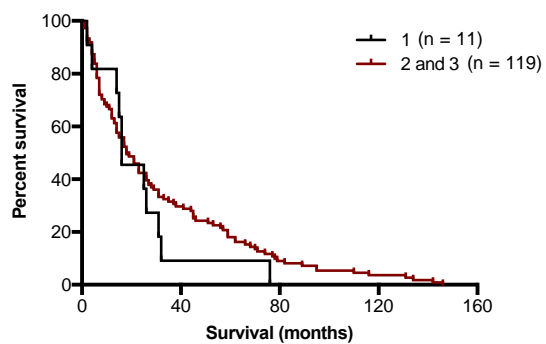

log-rank test  $p = 0.3944$

Supplement: Supplementary file 1 — Fig. S1. (A) Overall survival of HNSCC cases (n = 130) from the London Health Sciences Centre (LHSC), stratified by RICTOR IHC score (scores 0 & 1, versus 2 & 3). Cases scored as having RICTOR expression of 2 or 3 (n = 119) are represented in red. (B) Disease‐free survival of HNSCC cases (n = 130), stratified by RICTOR IHC score (scores 0 & 1, versus 2 & 3). Cases scored as having RICTOR expression of 2 or 3 (n = 119) are represented in red. [file MOL2-13-2160-s001.pdf]

RICTOR

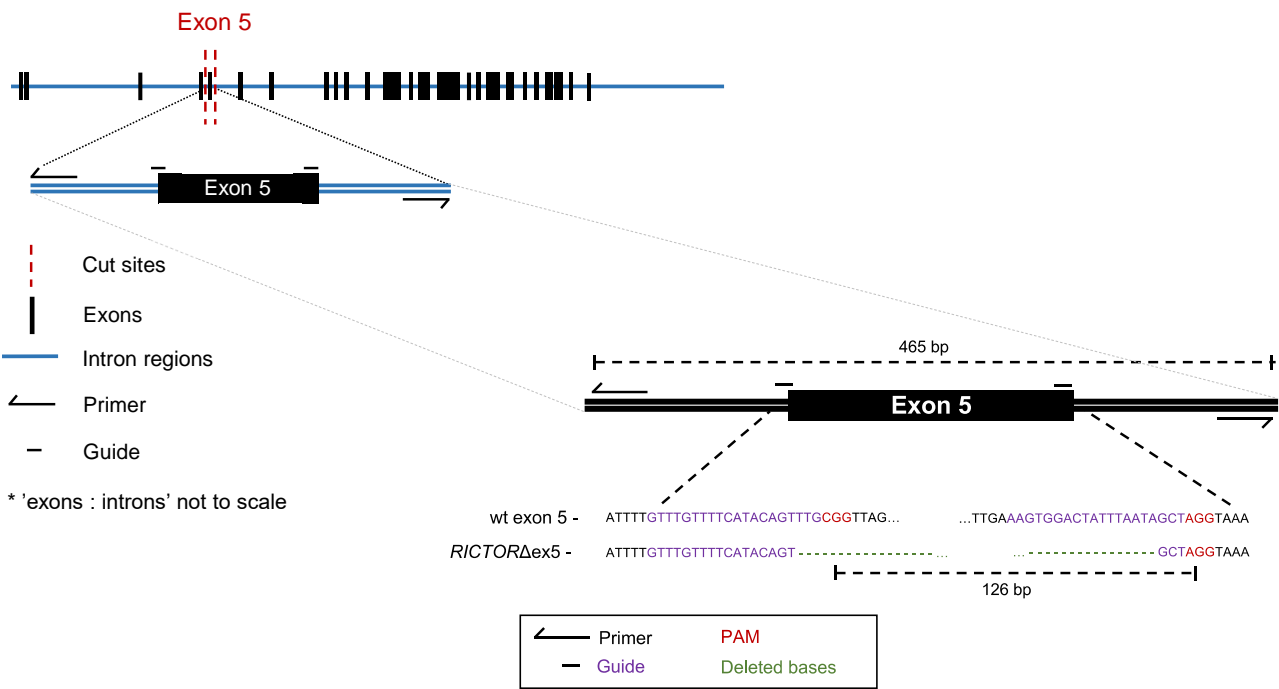

Supp. Fig 2  
 Ruicci KM *et al.*, 2018

Supplement: Supplementary file 2 — Fig. S2. Schematic illustrating design of single‐guide RNAs and primers for CRISPR/Cas9‐mediated deletion of exon 5 of RICTOR. [file MOL2-13-2160-s002.pdf]

**A. *RICTOR***

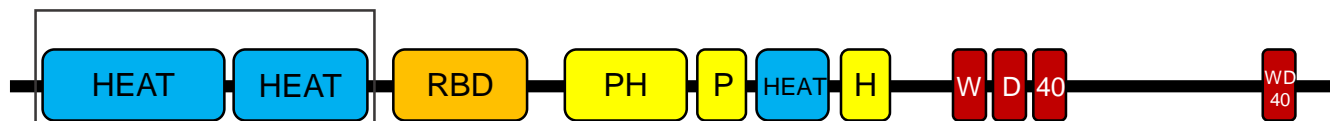

**B. *mTOR***

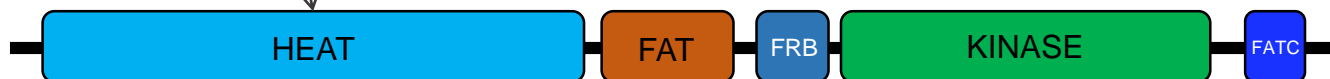

Supplement: Supplementary file 3 — Fig. S3. (A) Schematic representation of predicted domains of the human RICTOR gene. (B) Schematic representation of mTOR domains, with putative interacting HEAT domains of RICTOR and mTOR shown. Domain sizes not shown to scale. Adapted from Zhou P, et al., J Comp Bio, 2015. [file MOL2-13-2160-s003.pdf]

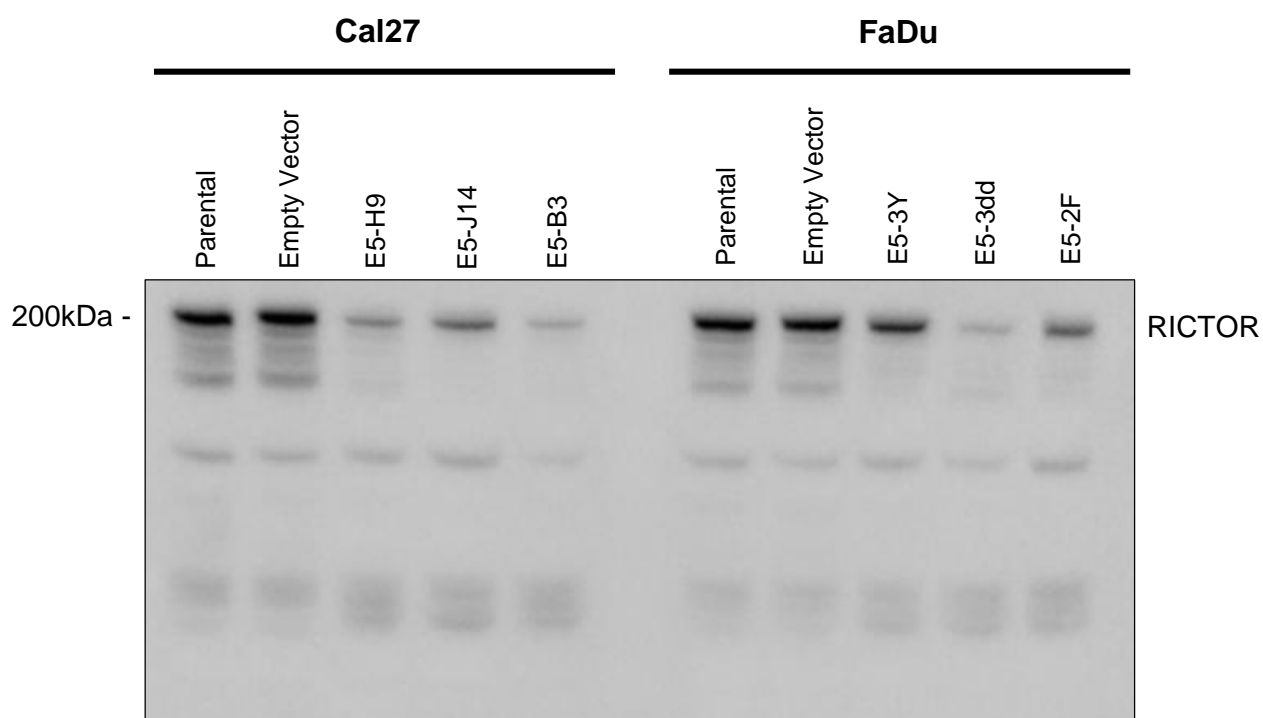

**Supp. Fig 4**  
**Ruicci KM *et al.*, 2018**

Supplement: Supplementary file 4 — Fig. S4. Immunoblot of RICTOR expression in parental and putative RICTOR knockout cell lines (E5‐XX lines). Full‐length gel shown in order to evaluate the presence of any truncated proteins forming following RICTOR exon 5 deletion. [file MOL2-13-2160-s004.pdf]

**A.**

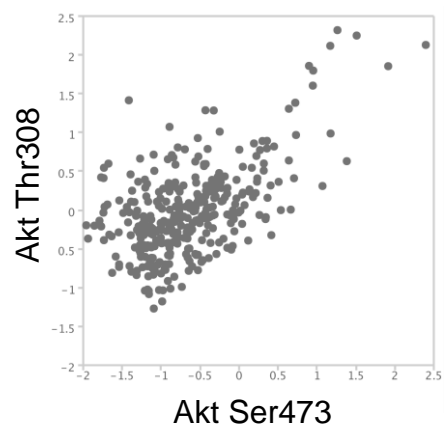

**B.**

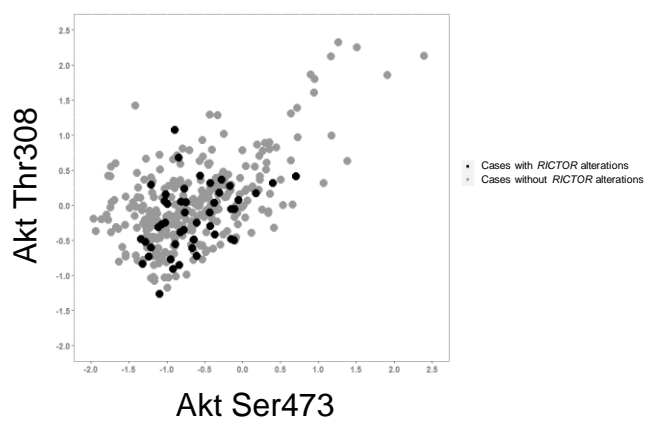

**C.**

**FaDu**

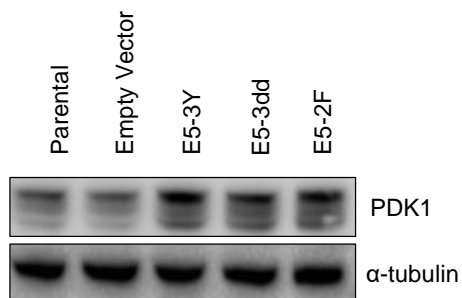

**Cal27**

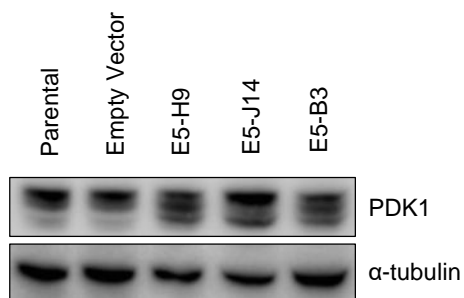

Supplement: Supplementary file 6 — Fig. S6. (A) Correlation between abundance of Akt (Thr308) and Akt (Ser473) in HNSCC primary tumor samples curated by The Cancer Proteome Atlas (TCPA). (B) Correlation between abundance of Akt (Thr308) and Akt (Ser473) in HNSCC primary tumor samples curated by TCPA, in relation to the presence or absence of RICTOR aberrations, as determined by TCGA. (C) Immunoblot of PDK1 expression in parental and RICTOR knockout cell lines (E5‐XX lines). [file MOL2-13-2160-s006.pdf]
