# Supplementary material for: Disruption of the RICTOR/mTORC2 complex enhances the response of head and neck squamous cell carcinoma cells to PI3K inhibition
Source: Mol Oncol. 2019 Aug 28;13(10):2160–77. doi: 10.1002/1878-0261.12558 (PMC6763779; doi:10.1002/1878-0261.12558)
Supplement: Supplementary file 5 — Fig. S5. Sequencing alignments for (A) FaDu and (B) Cal27 cell lines. RICTOR knockout cell lines underwent Sanger Sequencing and were aligned to their parental counterparts, revealing deletions of variable sizes in the RICTOR gene sequence spanning exon 5. The wild‐type gene sequence is at the top of each panel, indicated in bold text. [file MOL2-13-2160-s005.pdf]

A. FaDu

```
FaDu       1  ACTTGTATTTTGTTTGTTTTCATACAGTTTGCGGTTAGCTTTATTAAATGAAGCAAAAGA
E5-3Y      1  ACTTGTATTTTGTTTGTTTTCATACAG---------------------------------
E5-3dd     1  ACTTGTATTTTGTTTGTTTTCATACAG---------------------------------
E5-2FT     1  ANNTNTNTTTTNTNNNTTNTNNNNCNNTTTGCGGTTAGCTTTATTAAATGAAGCAAAAGA
E5-2FB     1  GCT---------------------------------------------------------

FaDu      61  AGTGCGAGCAGCAGGGCTACGAGCGCTTCGATATCTCATCCAAGACTCCAGTATTCTCCA
E5-3Y     28  ------------------------------------------------------------
E5-3dd    28  ------------------------------------------------------------
E5-2FT    61  AGTGCGAGCAGCAGGGCTACGAGCGCTTCGATATCTCATCCAAGACTCCAGTATTCTCCA
E5-2FB     4  ------------------------------------------------------------

FaDu     121  GAAGGTGCTAAAATTGAAAGTGGACTATTTAATAGCTAGGTAAATTTCCTAGACTTGTTT
E5-3Y     28  --------------------------------TAGCTAGGTAAATTTCCTAGACTTGTTT
E5-3dd    28  --------------------------------TAGCTAGGTAAATTTCCTAGACTTGTTT
E5-2FT   121  GAAGGTGCTAAAATTGAAAGTGGNCTATT--TTAGCTAGGTAAATTTCCTAGACTTGTTT
E5-2FB     4  -------------------------------------AGGTAAATTTCCTAGACTTGTTT

FaDu     181  ATATATTTTTGAAATTTTGTGTTGAGTTTTTAGCANNCNNNCCATAAAGTGTATAGATTT
E5-3Y     56  ATATATTTTTGAAATTTTGTGTTGAGTTTTTAGCATGCCTGCCATAAAGTGTATAGATTT
E5-3dd    56  ATATATTTTTGAAATTTTGTGTTGAGTTTTTAGCATGCCTGCCATAAAGTGTATAGATTT
E5-2FT   179  ATATATTTTTGAAATTTTGTGTTGAGTTTTTAGCATGCCTNCCATAAAGTGTATAGATTT
E5-2FB    27  ATATATTTTTGAAATTTTGTGTTGAGTTTTTAGCATGCNTGCCATAAAGTGTATAGATTT
```

B. Cal27

```
Cal27      1  NNNNN------------------GNTTGCGGTTAGCTTTATTAAATGAAGCAAAAGAAGT
E5-J14     1  TTGTATTTTGTTTGTTTTCANACAG-----------------------------------
E5-H9      1  TAG---------------------------------------------------------
E5-B3T     1  TTGTATTTTGTTTGTTTTCATACAGTTGCGGTTAGCTTTATTAAATGAAGCAAAAGAAGT
E5-B3B     1  NNNNN------------------N------------------------------------

Cal27     43  GCGAGCAGCAGGGCTACGAGCGCTTCGATATCTCATCCAAGACTCCAGTATTCTCCAGAA
E5-J14    26  ------------------------------------------------------------
E5-H9      4  ------------------------------------------------------------
E5-B3T    61  GCGAGCAGCAGGGCTACGAGCGCTTCGATATCTCATCCAAGACTCCAGTATTCTCCAGAA
E5-B3B     7  ------------------------------------------------------------

Cal27    103  GGTGCTAAAATTGAAAGTGGACTATTTAATAGCTAGGTAAATTTCCTAGACTTGTTTATA
E5-J14    26  -----------N-------------------GNTAGGTAAATTTCCTAGACTTGTTTATA
E5-H9      4  --------------------------------CTAGGTAAATTTCCTAGACTTGTTTATA
E5-B3T   121  GGTGCTAAAATT-------------------GCTAGGTAAATTTCCTAGACTTGTTTATA
E5-B3B     7  ---------------------------------------------NNNNTTGTTNANA

Cal27    163  TATTTTTGAAATTTTGTGTTGAGTTTTTAGCATGCCTGCCATAAAGTGTATAGATTTGTA
E5-J14    56  TATTTTTGAAATTTTGTGTTGAGTTTTTAGCATGCCTGCCATAAAGTGTATAGATTTGTA
E5-H9     32  TATTTTTGAAATTTTGTGTTGAGTTTTTAGCATGCCTGCCATAAAGTGTATAGATTTGTN
E5-B3T   162  TATTTTTGAAATTTTGTGTTGAGTTTTTAGCATGCCTGCCATAAAGTGTATAGATTTGTA
E5-B3B    20  TATTTTTGA-ATTTGTGTTGAGTTTTTAGCATGCCTGCCATAAAGTGTATAGATTTGTA
```

Supp. Fig 5

Ruicci KM *et al.*, 2018
