# Supplementary material for: Disruption of the RICTOR/mTORC2 complex enhances the response of head and neck squamous cell carcinoma cells to PI3K inhibition
Source: Mol Oncol. 2019 Aug 28;13(10):2160–77. doi: 10.1002/1878-0261.12558 (PMC6763779; doi:10.1002/1878-0261.12558)
Supplement: Supplementary file 7 — Table S1. Antibodies used in this study. [file MOL2-13-2160-s007.pdf]

Supplementary Table 1. Antibodies used in this study.

Antibody	Company	Catalogue Number	Dilution
**RICTOR**	CST	9476	1:1000
**p110α**	CST	4249	1:1000
**α-tubulin**	CST	2125	1:1000
**pAKT (T308)**	CST	4056	1:1000
**AKT (pan)**	CST	4685	1:1000
**pS6 (S240/4)**	CST	5364	1:1000
**S6**	CST	2217	1:1000
**EGFR**	CST	4267	1:1000
**p-mTOR (S2448)**	CST	2971	1:1000
**mTOR**	CST	2972	1:1000
**pAKT (S473)**	CST	4060	1:1000
**NDRG1**	CST	9485	1:1000
**NDRG1 (T346)**	CST	5482	1:1000
**PDK1**	CST	13037	1:1000
**RICTOR**$^{+}$	abcam	ab70374	1:1000

CST, Cell Signaling Technology
$^{+}$ for IHC
