# Supplementary material for: Disruption of the RICTOR/mTORC2 complex enhances the response of head and neck squamous cell carcinoma cells to PI3K inhibition
Source: Mol Oncol. 2019 Aug 28;13(10):2160–77. doi: 10.1002/1878-0261.12558 (PMC6763779; doi:10.1002/1878-0261.12558)
Supplement: Supplementary file 8 — Table S2. Clinical and pathological characteristics of 130 patients with HNSCC and association with RICTOR expression. [file MOL2-13-2160-s008.pdf]

Supplementary Table 2. Clinical and pathological characteristics of 130 patients with HNSCC and association with RICTOR expression

Clinicopathological variables		n (%)	RICTOR		
			Negative (0) / Weak, incomplete (1)	Moderate, incomplete (2) / strong, near complete (3)	*p* value
Age	<60	69 (53.1)	4	65	0.3461
	>=60	61 (47.9)	7	54	
Gender	Male	99 (76.2)	8	91	0.723
	Female	31 (23.8)	3	28	
Site	Tonsil	92 (70.8)	3	89	0.0018 *
	Base of Tongue	24 (18.5)	4	20	
	Other	14 (10.8)	4	10	
T Classification	1	31 (23.8)	2	29	0.8377
	2	48 (36.9)	4	44	
	3	28 (21.5)	2	26	
	4	23 (17.7)	3	20	
N Classification	0	20 (15.4)	1	19	0.7342
	1	22 (16.9)	2	20	
	2	78 (60.0)	7	71	
	3	10 (7.7)	0	10	
Overal Stage	1	6 (4.6)	0	6	0.8631
	2	9 (7.0)	1	8	
	3	19 (14.6)	2	17	
	4	96 (73.8)	8	88	
Smoking	never smokers	28 (21.5)	4	24	0.686
	1-9 py	8 (6.2)	1	7	
	10-19 py	13 (10.0)	1	12	
	>20 py	75 (57.7)	5	70	
	Unknown	6 (4.6)	0	6	
Alcohol (drinks/wk)	< 21 drinks	88 (67.7)	7	81	0.7378
	>21 drinks	38 (29.2)	4	34	
	Unknown	4 (3.0)	0	4	
Recurred	No	104 (80.0)	9	95	0.99
	Yes	26 (20.0)	2	24	
HPV	Negative	43 (47.8)	1	42	0.618
	Positive	46 (51.1)	3	44	

* Significant features; $p < 0.05$
